# Large Language Models May Help Patients Understand Peer-Reviewed Scientific Articles About Ophthalmology: Development and Usability Study

**DOI:** 10.2196/59843

**Published:** 2024-12-24

**Authors:** Reza Kianian, Deyu Sun, William Rojas-Carabali, Rupesh Agrawal, Edmund Tsui

**Affiliations:** 1 Stein Eye Institute Department of Ophthalmology David Geffen School of Medicine Los Angeles, CA United States; 2 Nanyang Technological University Lee Kong Chian School of Medicine Singapore Singapore; 3 Tan Tock Seng Hospital National Healthcare Group Eye Institute Singapore Singapore; 4 National Healthcare Group Programme for Ocular Inflammation & Infection Translational Research Singapore Singapore

**Keywords:** uveitis, artificial intelligence, ChatGPT, readability, peer review, large language models, LLMs, health literacy, patient education, medical information, ophthalmology

## Abstract

**Background:**

Adequate health literacy has been shown to be important for the general health of a population. To address this, it is recommended that patient-targeted medical information is written at a sixth-grade reading level. To make well-informed decisions about their health, patients may want to interact directly with peer-reviewed open access scientific articles. However, studies have shown that such text is often written with highly complex language above the levels that can be comprehended by the general population. Previously, we have published on the use of large language models (LLMs) in easing the readability of patient-targeted health information on the internet. In this study, we continue to explore the advantages of LLMs in patient education.

**Objective:**

This study aimed to explore the use of LLMs, specifically ChatGPT (OpenAI), to enhance the readability of peer-reviewed scientific articles in the field of ophthalmology.

**Methods:**

A total of 12 open access, peer-reviewed papers published by the senior authors of this study (ET and RA) were selected. Readability was assessed using the Flesch-Kincaid Grade Level and Simple Measure of Gobbledygook tests. ChatGPT 4.0 was asked “I will give you the text of a peer-reviewed scientific paper. Considering that the recommended readability of the text is 6th grade, can you simplify the following text so that a layperson reading this text can fully comprehend it? - Insert Manuscript Text -”. Appropriateness was evaluated by the 2 uveitis-trained ophthalmologists. Statistical analysis was performed in Microsoft Excel.

**Results:**

ChatGPT significantly lowered the readability and length of the selected papers from 15th to 7th grade (*P*<.001) while generating responses that were deemed appropriate by expert ophthalmologists.

**Conclusions:**

LLMs show promise in improving health literacy by enhancing the accessibility of peer-reviewed scientific articles and allowing the general population to interact directly with medical literature.

## Introduction

Health literacy is pivotal for empowering individuals to make informed health decisions, navigate the health care system, and manage their well-being. It bridges the gap between complex medical information and patient understanding, thus playing a crucial role in enhancing public health outcomes and reducing health disparities [1]. Readability in clinical medicine refers to the reading level required to fully comprehend the information presented in a body of medical text. The health literacy of the United States’ general population is poor, as the average person cannot comprehend text beyond an eighth-grade level, a limitation exacerbated among patients with Medicare or Medicaid [2]. Contributing to the rapid and unsustainable expansion of annual health care costs in the United States, poor health literacy has been shown to be detrimental to the nation’s general health [3]. A wealth of published work exists on the association between poor health literacy and more hospitalizations, inadequate follow-up, underuse of preventative care, poor medication compliance, and increased mortality [3,4].

To address these disparities, the American Medical Association (AMA) and the National Institutes of Health (NIH) recommend that the readability of patient-targeted health information be equivalent to a sixth- to eighth-grade level [2,5]. This is particularly crucial in the age of the internet, as over half of the US population browses health information on the internet to learn about their conditions and their management [6].

Numerous studies have demonstrated that the readability of health information found on the internet, including content published by academic institutions, is often poor and notably more complex than the recommended levels [7-10]. With the recent surge and growing popularity of large language models (LLMs), such as ChatGPT (OpenAI), some investigators have begun exploring the possibility of using this tool to enhance the readability and accessibility of health information for patients [11-18]. Our team recently conducted a study showcasing ChatGPT’s ability to rewrite existing health information with poor readability into documents with reading levels that align with the recommendations of the AMA and NIH [13].

Recent studies have shown the readability of scientific texts to be getting worse over time with a study showing the average reading level required to understand papers and their abstracts to be as high as 17th grade (college graduate level) [19,20]. Building on our previous research, which demonstrated the efficacy of ChatGPT in addressing health literacy challenges, our objective was to assess whether this technology could successfully transform the complex language found in scientific papers into more accessible health information. This evaluation specifically aimed to cater to laypersons seeking medical advice on topics related to uveitis.

## Methods

### Overview

To conduct this study, we identified 12 open access papers on various uveitis-related topics published by the 2 senior authors of this study (ET and RA). The papers covered several available study types, such as case reports, imaging studies, prospective studies, retrospective studies, and review papers. The readability of these articles was assessed using 2 validated and commonly used tools in the literature, that is, Flesch-Kincaid Grade Level (FKGL) and Simple Measure of Gobbledygook (SMOG). There are other readability assessment tools, such as the Gunning Fog index, Coleman-Liau index, and automated readability index, available. However, the FKGL and the SMOG tools are the 2 most commonly used readability assessment tools that we identified in our literature review. Total words, sentences, syllables, and polysyllabic words are used in the formulas underpinning these 2 readability tools. A calculator (readabilityformulas website) was used. We then asked ChatGPT 4.0 the following prompt: “I will give you the text of a peer-reviewed scientific paper. Considering that the recommended readability of the text is 6th grade, can you simplify the following text so that a layperson reading this text can fully comprehend it? - Insert Manuscript Text -”. Appropriateness was evaluated similarly to criteria previously published by 2 fellowship-trained uveitis physicians (ET and RA) who authored the papers selected for this study [13,21]. Responses were marked as “appropriate” or “inappropriate” based on the authors’ clinical experiences and knowledge of the literature. An appropriate response was one that accurately simplified the entire text of the article without including any information that would be deemed as false or inaccurate. An inappropriate response was one that either included false or inaccurate information or included information that was not intended by the authors of the study. If there were disagreements between the 2 authors on the appropriateness of a generated response, a third fellowship-trained uveitis physician would be asked for an independent opinion. Statistical analysis was performed using Microsoft Excel (version 2401). Paired-sample *t* test was used to compare the average readability and the word count obtained from the original text of the included articles with the generated responses by ChatGPT with the recommended sixth-grade readability levels. Descriptive statistics were used to represent the rest of the data.

### Ethical Considerations

Human participants and their associated data were not used in this study. Therefore, no informed consents, statements on language waivers, and privacy, and confidentiality statements are necessary.

## Results

ChatGPT was able to rewrite scientific articles into an appropriate body of text with significantly improved reading levels (*P*<.001). The average reading level of the original text was around 15th grade, and it was reduced to a seventh-grade reading level. This improvement was present across all study types analyzed. The output generated by ChatGPT was also found to be appropriate and accurately represent the data presented in the original text in a simple format without any medical mistakes. The average word count for the original papers was 2882 (SD 1348) words, and ChatGPT responses had an average count of 417 (SD 78) words (*P*<.001). The readability and appropriateness of each response, along with the original readability of the paper and study types are documented in Table 1.

**Table 1:** Selected open access research articles and their corresponding readability and appropriateness.

Title	Type	Original FKGL^a^	ChatGPT FKGL	Original SMOG^b^	ChatGPT SMOG	Appropriateness (yes or no)
Delayed onset anterior uveitis and macular edema after cessation of pembrolizumab	Case report	16.17	7.4	14.65	6.57	Yes
Post typhoid fever neuroretinitis with serous retinal detachment and choroidal involvement-A case report	Case report	16.41	4.72	14.84	4.57	Yes
Choroidal Vascularity Index (CVI)—A Novel Optical Coherence Tomography Parameter for Monitoring Patients with Panuveitis?	Imaging study	16.34	8.3	14.79	8.21	Yes
Quantification of Anterior Chamber Cells in Children with Uveitis Using Anterior Segment Optical Coherence Tomography	Imaging study	16.92	8.38	15.25	8.9	Yes
Evaluation of Retinal Vascularity Index in Patients with COVID-19: A Case–Control Study	Prospective study	15.19	7.26	14.05	7.31	Yes
Choroidal structural changes in preterm children with and without retinopathy of prematurity	Prospective study	12.12	7.33	11.96	7.2	Yes
Implementation of a vision-screening program in rural northeastern United States	Prospective study	14.91	7.37	14.11	6.62	Yes
Choroidal vascularity index as a measure of vascular status of the choroid: Measurements in healthy eyes from a population-based study	Prospective study	14.26	7.23	12.72	7.27	Yes
Factors affecting final functional outcomes in open‐globe injuries and use of ocular trauma score as a predictive tool in Nepalese population	Retrospective study	14.04	8.29	13.65	8.41	Yes
Prognostic factors for vision outcome after surgical repair of open globe injuries	Retrospective study	12.9	7.57	13.07	8.01	Yes
Multimodal imaging in pediatric uveitis	Review paper	17.83	9.17	16.12	9.59	Yes
Recent advances in the treatment of juvenile idiopathic arthritis–associated uveitis	Review paper	24.11	7.08	20.41	7.17	Yes
Average (SD)		15.93 (3.07)	7.51 (1.08)	14.64 (2.15)	7.49 (1.29)	
*P* value		<.001	<.001	<.001	<.001	

^a^FKGL: Flesch-Kincaid Grade Level.

^b^SMOG: Simple Measure of Gobbledygook.

## Discussion

### Principal Findings

In summary, ChatGPT lowered the readability of open access and peer-reviewed science articles by 8 grade points from 15th grade to 7th grade (*P*<.001). Since the introduction of LLMs like ChatGPT just over a year ago, there has been increasing interest in the use of such artificial intelligence (AI) models in health care and clinical education. In addition to generating patient-targeted health information, investigators have explored the use of LLMs in medical education, guiding patients postoperatively, and answering common questions [22-26]. The authors of this study were some of the first to examine the role of ChatGPT in producing readable health care information or rewriting existing patient-targeted information. This analysis is an extension of such a study.

Previously, we found mixed results when asking ChatGPT to produce patient-targeted health information regarding uveitis and surgical management of glaucoma. In 1 study, we illustrated the superiority of ChatGPT to Bard, Google’s AI, and demonstrated that ChatGPT produced educational material for uveitis at the recommended sixth-grade reading level [13]. ChatGPT was also able to convert already existing health information found on the internet with a readability of 11th grade into significantly more readable content with a reading level of eighth grade. In another study, however, while Kianian et al [12] demonstrated the reliability of ChatGPT in assessing the quality of health information targeted to the layperson, they found that ChatGPT was not able to produce highly readable information on the surgical management of glaucoma.

The findings of this study align more with our previous study on ChatGPT and uveitis. When asked to rewrite peer-reviewed scientific papers with complex texts and a readability of 15th grade into easier-to-understand information for a layperson, ChatGPT was able to produce appropriate responses with a readability of seventh grade. These findings are promising since this can potentially improve the comprehension of peer-reviewed open access scientific articles for the general population while maintaining accurate information. Before the introduction of LLMs to the population, patients may have had difficulty comprehending highly technical and difficult-to-understand scientific articles. Previous studies support the positive influence of improved medical literature comprehension and health knowledge on trust in scientific bodies and the enhanced management of conditions by patients [27,28]. Therefore, the use of ChatGPT by patients may contribute meaningfully to public trust in the scientific process.

Patients may benefit from directly engaging with peer-reviewed research as their ability to comprehend research may enhance their care on multiple fronts [29]. Extensive research has identified that understanding the rationale behind medical decisions is a significant factor in improving patients’ adherence to their medical regimen across specialties [30]. Furthermore, patients may become more well-informed when faced with inaccurate and misleading controversies, such as the link between vaccines and autism or the vast amount of misinformation present on the COVID-19 pandemic [29]. To combat health misinformation, Swire-Thompson and Lazer [31] argue for improved health literacy, using the internet collaboratively with physicians and stronger signal of source quality. The ability of the general public to directly interact with clinical trials and peer-reviewed scientific articles may aid in empowering patients in making better-informed decisions regarding their health.

### Limitations

There are limitations to this analysis and its application. Although we checked for the appropriateness of the information generated by ChatGPT, studies have shown that ChatGPT can sometimes produce inaccurate information [32,33]. Therefore, we urge patients to not solely base their health education on ChatGPT rewrite of scientific articles, rather we recommend that they consult their physicians regarding their health conditions. Second, it is possible that 1 article may not by itself provide context or provide all the information needed to make sound decisions regarding care. Hence, patients must study various trusted sources of information to make informed decisions. Furthermore, we are not aware if the appropriateness of the responses would be affected negatively if patients ask for a follow-up to make the generated responses even shorter. Third, although readable content is essential for comprehension, future studies may recruit patients in well-designed prospective studies to assess the true potential of ChatGPT in the comprehension of medical education and their decision-making. Fourth, the version of ChatGPT (version 4.0), which was used in this study, requires a monthly subscription fee. However, previous studies have demonstrated the ability of the free version of ChatGPT (version 3.5) to generate and also rewrite patient-targeted health information in a language with significantly improved readability scores [34,35]. Therefore, we suspect that ChatGPT-3.5 could also be helpful to patients. Fifth, only peer-reviewed scientific articles on topics mostly around uveitis written by the 2 senior authors of this study were analyzed in this investigation. It is important for the readers to avoid making generalizations from the findings of our study toward all published scientific articles in the field of ophthalmology or, more broadly, medicine. Finally, although we were careful to choose open access articles that are coauthored by the senior authors of this paper, we do recognize that further evaluation and oversight are needed to determine the legality and the ethics of entering copyrighted material, or any form of intellectual property, into LLMs such as ChatGPT.

### Future Work

In this investigation, we only chose peer-reviewed open access articles that have been coauthored by the senior authors of this study for several reasons. First, given concern regarding the ethics of inputting text from peer-reviewed papers written by other authors not involved in this study, we decided to use papers that are both open access as well as written by the senior authors of this study. This choice, we believe, also allowed for a better judgment when assessing the appropriateness of ChatGPT’s generated summary of the inputted text.

As the popularity of LLMs, such as ChatGPT, rises among health care professionals and patients across the world, the application of AI, such as ChatGPT, in simplifying the text of additional highly impactful articles for the general population should be investigated. A recent study by Sener et al [36] has identified the top 50 most-cited articles within the field of uveitis, and another article by Ohba et al [37] in the *Journal of American Medical Association* (*JAMA*) has identified the 100 most frequently cited articles in the field of ophthalmology. Sener et al [36] use a unique approach in identifying articles that are not only highly cited in scientific journals, but also those that are cited frequently across social media platforms [36]. Given the exposure of the general public to such articles, the methodology of our study could be applied to the identified peer-reviewed papers.

### Conclusions

In conclusion, our study demonstrates the exciting potential of LLMs, demonstrated by ChatGPT, in giving the general population the power and the tools needed to tackle the challenge of engaging directly with peer-reviewed open access scientific articles. In this study, ChatGPT was able to appropriately rewrite poorly readable scientific papers into an appropriate seventh-grade response. We advocate caution, however, as LLMs are integrated more and more into the daily life of the general population, and we emphasize the importance of consultation with health care providers before making health decisions.

## Abbreviations

AI: artificial intelligence
AMA: American Medical Association
FKGL: Flesch-Kincaid Grade Level
LLM: large language model
NIH: National Institutes of Health
SMOG: Simple Measure of Gobbledygook
